# Supplementary material for: Spin-exciton coupling modified by interfacial magnetic interactions in a van der Waals heterostructure
Source: Nat Commun. 2026 Feb 10;17:2551. doi: 10.1038/s41467-026-69389-x (PMC13000211; doi:10.1038/s41467-026-69389-x)
Supplement: Supplementary file 1 — Supplementary Information [file 41467_2026_69389_MOESM1_ESM.pdf]

Supplementary Materials

Spin-exciton coupling modified by interfacial magnetic interactions in a van der Waals heterostructure

Weican Lan[1,†], Chaocheng Liu[1,†,*], Yajuan Feng[1,†], Ruiqi Liu[1], Yafei Chu[1], Lu Cheng[1], Chao Wang[1], Huijuan Wang[2], Minghui Fan[3], Zixun Zhang[3], Yuran Niu[4], Jheng-Cyuan Lin[5], Francesco Maccherozzi[5], Hengli Duan[5,*], Wensheng Yan[1,*]

[1]National Synchrotron Radiation Laboratory, University of Science and Technology of China, Hefei 230026, China

[2]Experimental Center of Engineering and Material Science, University of Science and Technology of China, Hefei 230026, China

[3]Hefei National Research Center for Physical Sciences at Microscale, University of Science and Technology of China, Hefei 230026, China

[4]MAX IV Laboratory, Lund University, Lund 22100, Sweden

[5]Diamond Light Source, Harwell Science and Innovation Campus, Didcot OX11 0DE, United Kingdom

[†]These authors contributed equally to this work

[*]Corresponding authors:
Chaocheng Liu — Email: chaochengliu@ustc.edu.cn
Hengli Duan — Email: hengli.duan@diamond.ac.uk
Wensheng Yan — Email: ywsh2000@ustc.edu.cn

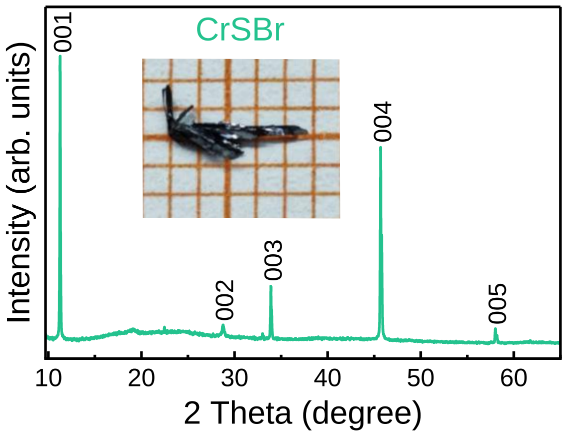

Fig. S1. Structural phase of CrSBr. XRD characterization of CrSBr single crystal. The inset shows an optical image of a typical bulk crystal.

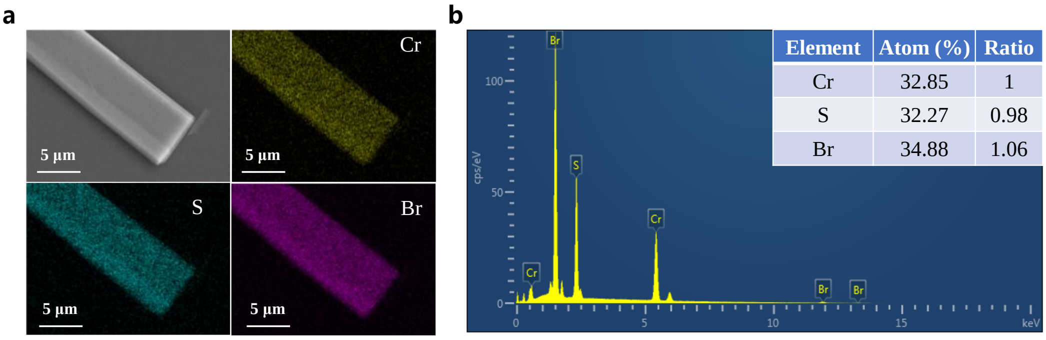

Fig. S2. Element distribution of CrSBr. a, SEM image and corresponding EDS elemental mappings of CrSBr. Scale bar, 5 µm. **b**, EDS spectrum of CrSBr.

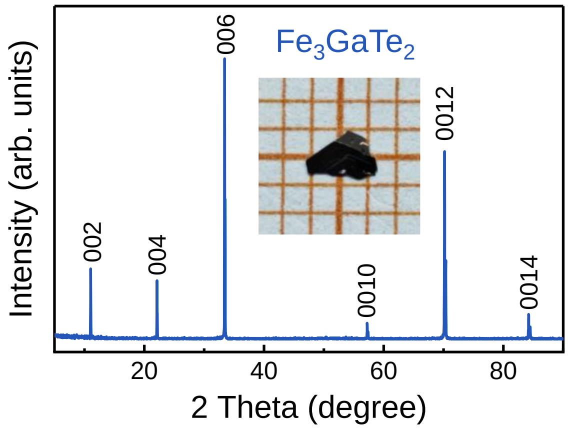

Fig. S3. Structural phase of FGT. XRD characterization of FGT single crystal. The inset shows an optical image of a typical bulk crystal.

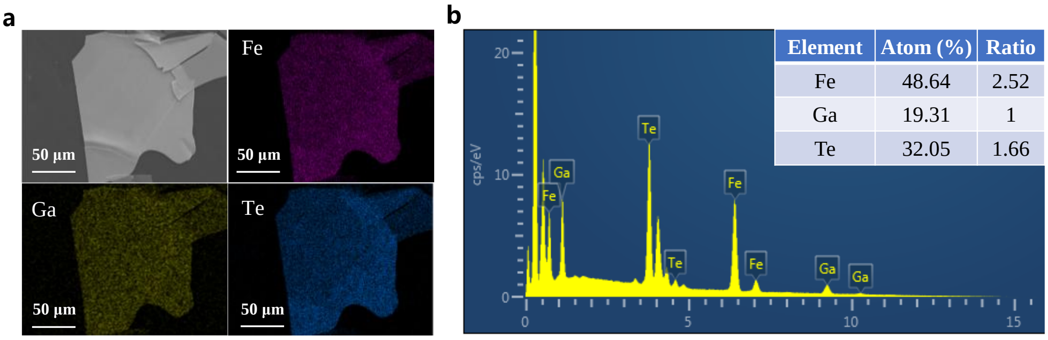

Fig. S4. Element distribution of FGT. a, SEM image and corresponding EDS elemental mappings of FGT. Scale bar, 5 µm. **b**, EDS spectrum of FGT.

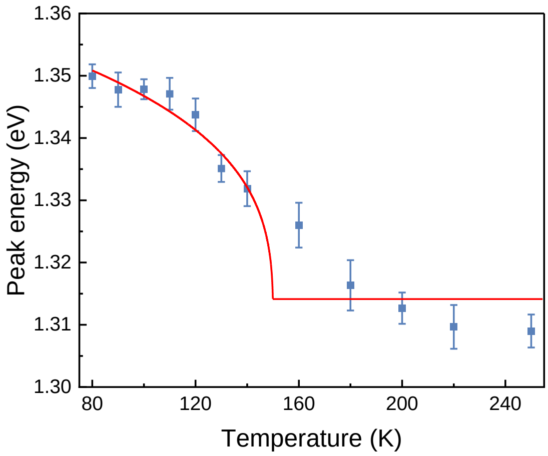

Fig. S5. Magnetic-order-dependent exciton fitting. Variations of the PL peak positions for CrSBr/FGT under different temperatures. The red solid curve is a best fit to the power law function $P^{i}(PL\ intensity) \propto \left[a + b(T - T_N)^{\beta}\right]^2$.

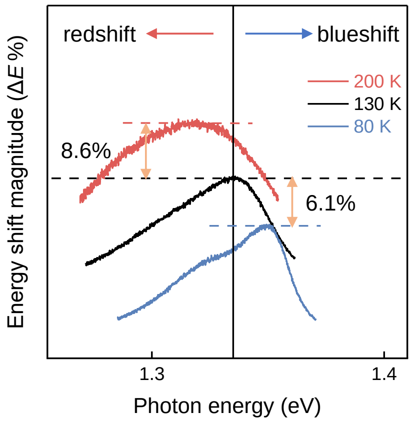

Fig. S6. Temperature-dependent modulation of exciton energy relative to the excitonic linewidth. The relative energy shift, expressed as a percentage of the excitonic linewidth (~100 meV), is plotted for three representative temperatures.

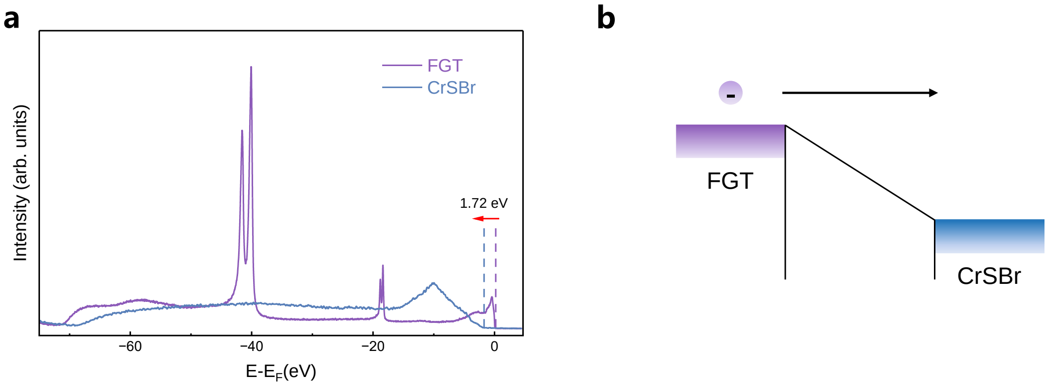

Fig. S7. Band information for heterostructure. a, UPS spectra of FGT and CrSBr. **b**, Charge transfer diagram.

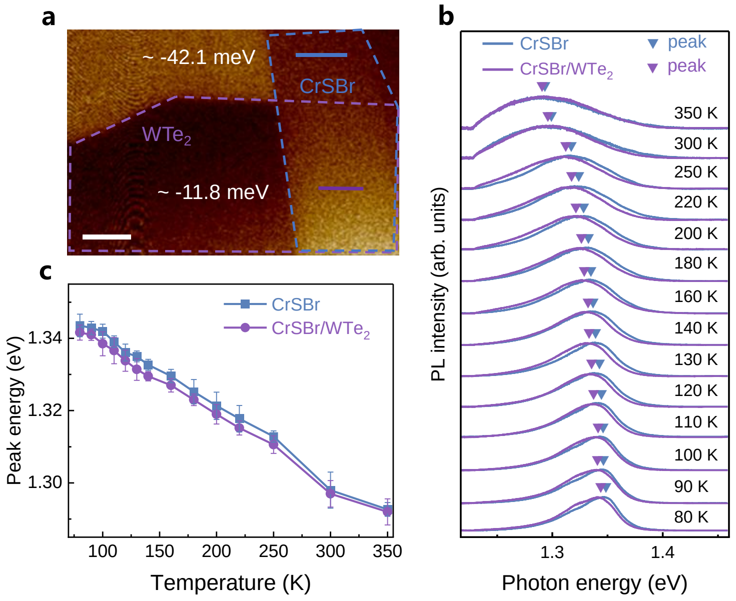

Fig. S8. KPFM and temperature-dependent PL for CrSBr/WTe_2. a, KPFM topography and work function plots of CrSBr/WTe_2 heterostructures. The blue and purple dashed lines mark the areas of CrSBr and WTe_2, respectively. Scale bar, 5 μm. **b**, Temperature-dependent PL spectra for pristine CrSBr and CrSBr/WTe_2. The triangles correspond to the energy position of their PL peaks. **c**, Variations of the PL peak positions for pristine CrSBr and CrSBr/WTe_2 under different temperatures. Error bars represent the standard deviation of three measurements.

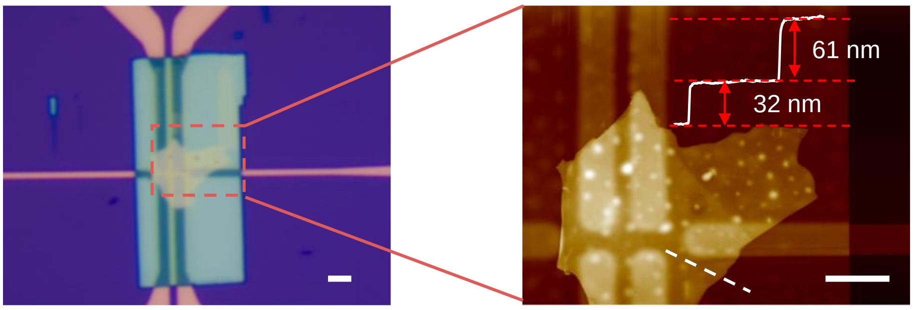

Fig. S9. Device structure. Optical image and atomic force microscopy of FGT/CrSBr heterostructure. Scale bar, 5 μm.

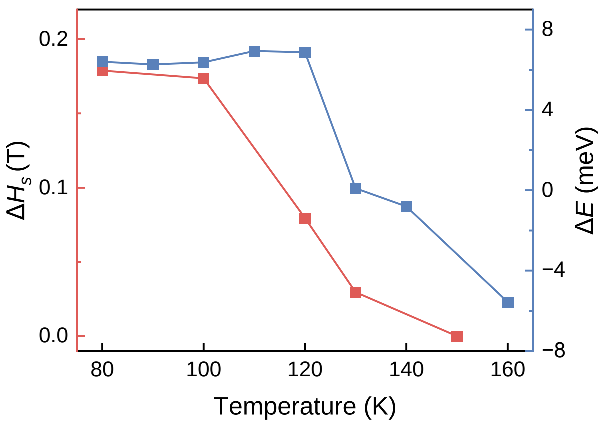

Fig. S10. Correlation between spin-flip and exciton energy. Temperature-dependent evolution relationship between the extracted spin-flip field and the exciton energy difference.

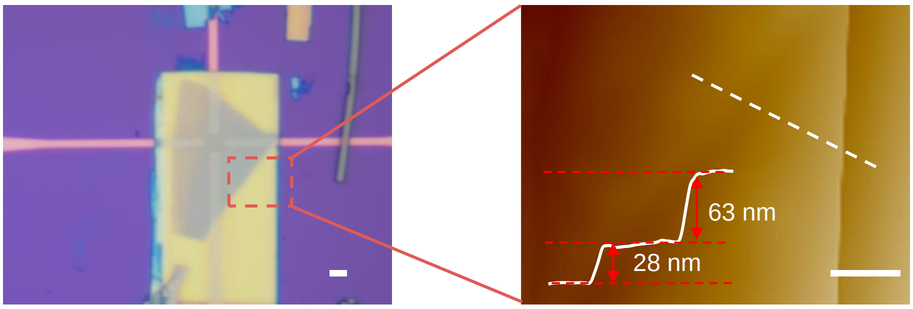

Fig. S11. Device structure. Optical image and atomic force microscopy of CrSBr/FGT heterostructure. Scale bar, 5 μm.

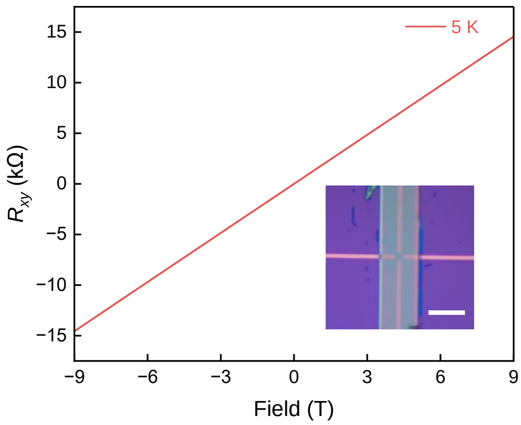

Fig. S12. Hall measurement of CrSBr. R_{xy} of the pristine CrSBr versus magnetic field at 5 K, measured with a perpendicular applied magnetic field. The inset shows an optical image of the CrSBr device. Scale bar, 20 μm.

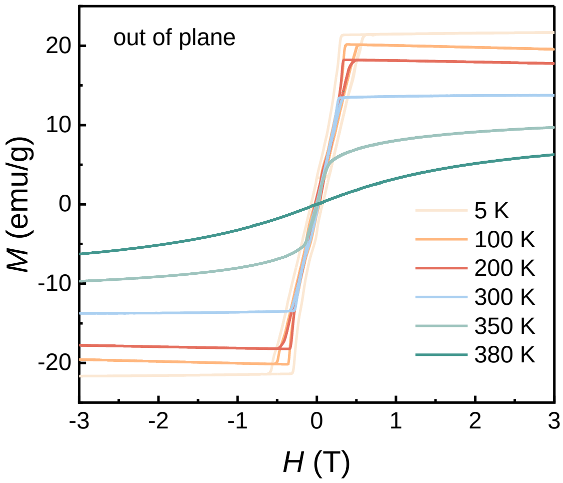

Fig. S13. Out-of-plane *M-H* for FGT. Field-dependent magnetization plot of FGT at varying temperatures, under a magnetic field along the out-of-plane of the crystal.

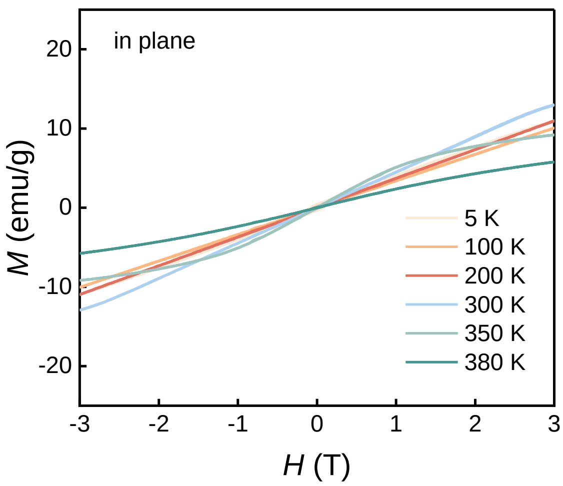

Fig. S14. In-plane *M-H* for FGT. Field-dependent magnetization plot of FGT at varying temperatures, under a magnetic field along the in-plane of the crystal.

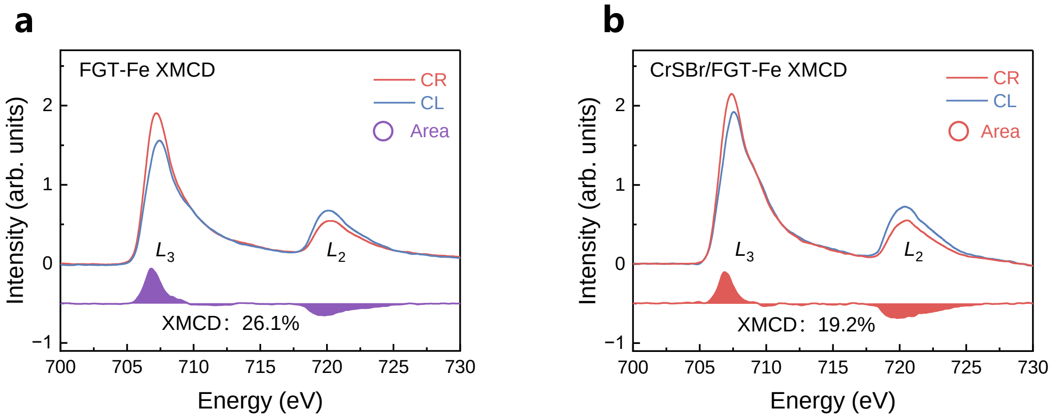

Fig. S15. XMCD signal. XMCD of Fe-*L* edge in FGT (**a**) and in CrSBr/FGT (**b**) extracted from the dark areas.

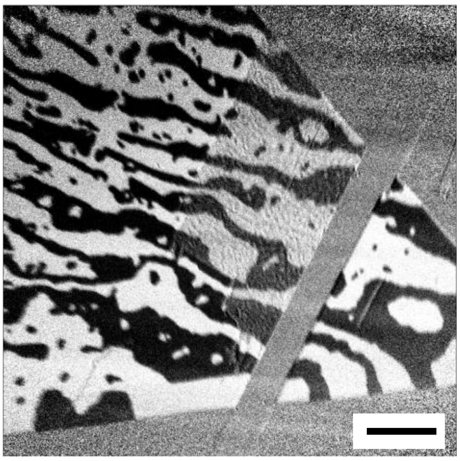

Fig. S16. XMCD-PEEM. Fe-*L* edge XMCD-PEEM image at 300 K. Scale bar, 4 μm.

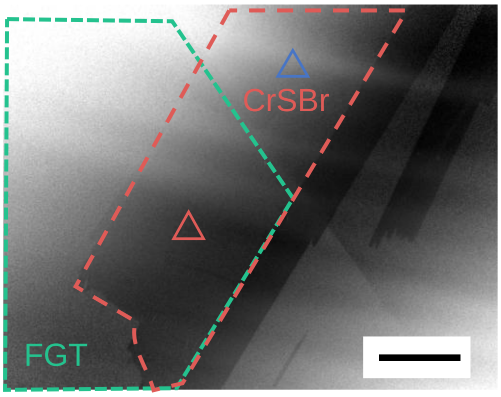

Fig. S17. XMLD-PEEM. Cr-*L* edge XMLD-PEEM image acquired with a 16° X-ray incidence angle relative to the sample surface. Scale bar, 4 μm. The triangle frames marked by blue and red respectively represent the pristine and heterostructure areas used for XMLD data extraction.

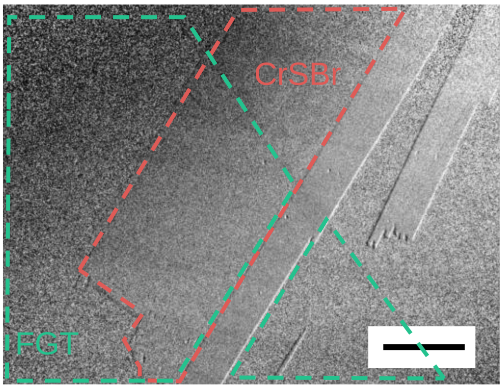

Fig. S18. XMCD-PEEM. Cr-*L* edge XMCD-PEEM image at 100 K. The red and green dashed lines mark the areas of CrSBr and FGT, respectively. Scale bar, 4 µm.

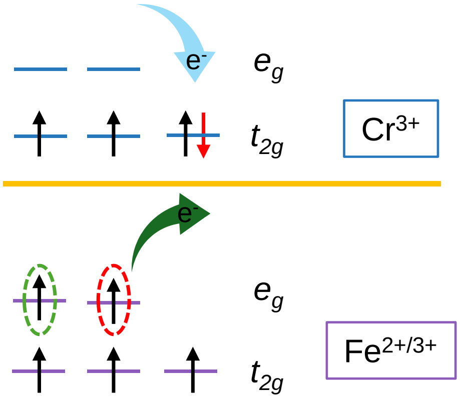

Fig. S19. Schematic diagram of charge transfer. Illustration of orbital-mediated charge transfer mechanism.

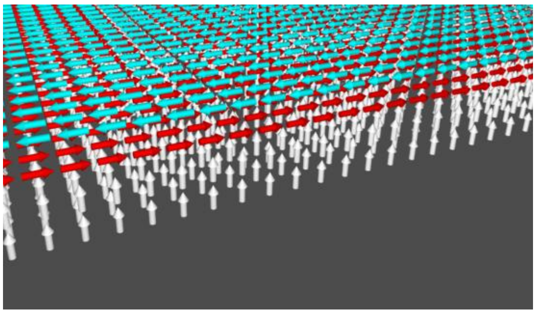

Fig. S20. Micromagnetic simulations. Schematic of the four-layer model, consisting of two out-of-plane ferromagnetic FGT layers and two in-plane antiferromagnetic FGT layers.

Table S1. Amount of electron transfer. DFT calculated electronic transfer in CrSBr/FGT heterostructure. The symbol "+" denotes electron gain, while "–" represents electron loss. Unit: e/$Å^2$.

CrSBr	FGT
+0.007	−0.007
